# Myocarditis following COVID-19 vaccination in adolescents: Cardiac magnetic resonance imaging study

**DOI:** 10.3389/fcvm.2022.978592

**Published:** 2022-10-05

**Authors:** Arthur Shiyovich, Ygal Plakht, Guy Witberg, Amichai Rotstein, Yaron Aviv, Maya Wiessman, Ran Kornowski, Ashraf Hamdan

**Affiliations:** ^1^Department of Cardiology, Rabin Medical Center, Faculty of Medicine, Tel-Aviv University, Tel Aviv, Israel; ^2^Faculty of Health Sciences, Ben-Gurion University, Soroka University Medical Center, Beer-Sheva, Israel; ^3^Department of Pediatric Cardiology, Schneider Children's Medical Center of Israel, Petach Tikva, Israel

**Keywords:** MRI, COVID-19, vaccine, myocarditis, adolescents

## Abstract

**Introduction:**

Vaccination-associated myocarditis was reported following COVID-19 vaccine initially among persons aged 16 or older and recently among adolescents aged 12–15.

**Objectives:**

To describe the clinical and cardiac magnetic resonance (CMR) characteristics of adolescents aged 12–15 with myocarditis following the administration of the BNT162b2 mRNA COVID-19 vaccine.

**Methods:**

CMR of adolescents (age 12–15) with a clinical diagnosis of myocarditis within 42 days following the first COVID-19 vaccine were analyzed.

**Results:**

A total of 182,605 adolescent were vaccinated, out of which 9 were diagnosed with clinically adjudicated myocarditis while CMR was performed in 5/9 patients (56%). Median age was 15 years (range 13–15), 4/5 (80%) males. All the patients we previously healthy. The ECG upon presentation was abnormal in 3/5 (60%) of patients. All cases were classified as clinically mild and no patient required inotropes or mechanical circulatory support treatment. The median follow-up time, for the 5-included patients, was 206 (IQR 192–229, range 179–233) days. During the follow-up, no re-admissions, deaths, or any other cardiac events have occurred.

The median time between the diagnosis to the CMR was 104 days (range 27–149). The median left ventricular ejection fraction was within normal range 65% (range 62–69). Native T1 was available in four patients, the local T1 value was increased in three of them. T2 values were available in two patients and were all within normal range. The median late gadolinium enhancement (LGE) was 2% (range 0–6%) with inferolateral wall being the most common location (3/5). The patterns of the LGE were as following: (i) mid-wall in 3 patients; (ii) epicardial in 1-patient. LGE in the pericardium was present in 2/5 patients with pericardial effusion present in 4/5 patients with a median diameter of 4 mm (range 3–5 mm) at end-systole.

**Conclusions:**

CMR findings and clinical course of adolescents with COVID-19 vaccination associated myocarditis, are similar to those of older patients, being relatively mild and potentially implying favorable outcomes.

## Introduction

Pfizer-BioNTech BNT162b2 messenger-RNA (mRNA) vaccines have demonstrated exceptional safety and real-world effectiveness in preventing severe disease and death from COVID-19. Concerns about vaccination-related myocarditis in young men were initially raised in Israel (1, 2). The Incidence of such myocarditis was highest among males aged 16–29 years, with a relatively favorable clinical course and mild cardiac magnetic resonance (CMR) imaging findings in initial reports (3, 4). Subsequently, mRNA COVID-19 vaccine was approved for adolescents aged 12–15 years and initial evidence of vaccine-associated myocarditis in this age group were published as well (5, 6). Our objective in the current report was to describe the clinical course and CMR imaging characteristics of adolescents aged 12–15 with myocarditis following the administration of BNT162b2 mRNA COVID-19 vaccine.

## Methods

### Study population

Data regarding 12–15 year old adolescents vaccinated between June 2nd, 2021 and November 30th, 2021 were obtained from Clalit Health Services (CHS) database. Potential myocarditis cases were identified throughout the period of 42 days after the administration of the first vaccine dose by ICD-9 codes. Subsequently, the diagnosis was adjudicated by cardiologists who carefully reviewed each patients' full-medical record. Follow-up data from the computerized medical record was performed until February 26th, 2022, as previously described (1).

This study was approved by the institutional review board and performed consistently with the Helsinki declaration.

### CMR imaging

CMR imaging was performed using either 1.5 T scanner (Ingenia; Philips Medical System) or 3 T scanner (Magnetom Vida; Siemens Healthineers), implementing standardized imaging protocols. CMR protocol comprised of multiplanar cine imaging for acquisition of cardiac function, volumes, and mass, and late gadolinium (LGE) for scar imaging. At 1.5 T scanner Balanced steady state free precession, single breath-hold modified inversion recovery Look-Locker (MOLLI) was used for T1 mapping and a navigator gated black blood prepared gradient spin-echo sequence was used for T2 mapping. At 3 T scanner, myocardial T1 mapping was performed using MOLLI sequence. Native T1 and T2 mapping, and postcontrast T1 mapping were acquired in a 3 short-axis slices (apical, mid-ventricular and basal).

Analysis of the CMR was performed as previously reported (3, 4). For 1.5T scanner abnormal native T1 and T2 values were defined as greater than 1,060 ms and greater than 57 ms; respectively (7) and for 3T scanner abnormal native T1 values were defined as greater than 1,105 ms (8).

### Statistical analysis

Descriptive statistical methods were applied in the current study. Baseline characteristics of the patients are presented as counts (%) for categorical variables and median (range) or mean (±standard deviation) for continuous variables, as appropriate.

## Results

A total of 182,605 adolescent were vaccinated during the study period, out of which 23 had an ICD-9 code of myocarditis. Following adjudication 9 adolescents were clinically confirmed to have a diagnosis of myocarditis. Throughout the study period CMR was performed in 5/9 patients (56%) who were included in the current study. Patient characteristics are presented in Table 1. Median age was 15 years (range 13–15), 4/5 (80% males). One patient presented following the first vaccine (3 days) and 4 after the second vaccine (median of 3 days). All the patients we previously healthy. The median temperature upon presentation was 37.8 (range: 36.5–38.6)°C. All patients presented with acute chest pain. The ECG upon presentation was abnormal in 3/5 (60%) of patients. The median value of the peak Troponin T level was 730 ng/L (IQR = 146–1,647) and the median C-reactive protein was 8.5 mg/dL (IQR = 4.2–14.2). The median left-ventricular ejection fraction, as evaluated by echocardiography at diagnosis, was 60% (range: 55–65%). All cases were classified as clinically mild and no patient required inotropes or mechanical circulatory support treatment. The median time of in-hospital stay was 4 days (range 2–6 days). The median follow-up time, for the five-included patients, was 206 (IQR 192–229, range 179–233) days. During the follow-up, no re-admissions, deaths, or any other cardiac events have occurred.

**Table 1:** Clinical characteristics and CMR findings of the study patients.

**Age**	**Sex**	**past medical history**	**Symptoms**	**ECG**	**Peak Troponin (ng/L)**	**Time from vaccine and symptoms (days) **	**Time from vaccine and CMR (days) **	**WMA**	**LVEDV (ml)**	**LESVD (ml)**	**LVEF (%)**	**T1 local (ms)**	**T1 Global (ms)**	**T2 local (ms)**	**T2 global (ms)**	**LGE localization**	**LGE %**	**LGE pattern**	**Pericardial enhance-ment**	**Pericardial effusion**	**Pericardial diameter**
15	M	None	Chest pain	Normal	146	3	149	N	133	42	69			51	49	–	0	-	N	N	-
15	M	None	Chest pain	Normal	730	5	107	N	144	50	65	1,042	1,044			Infero-lateral (medial)	2	Mid-wall	Y	Y	
15	M	None	Chest pain	Diffuse STE	1,190	3	104	N	132	51	62	1,076	1,075	53	48	Inferior, infero-lateral (basal/medial) and lateral (apical)	3	Mid-wall	N	Y	3
14*	F	None	Chest pain	Diffuse STE	350	2	95	N	138	49	68	1,313	1,251	NA	NA	Infero-lateral, inferoseptal (basal/medial)	2	Mid-wall	N	Y	4
13	M	None	Chest pain	Inferior STE	1,647	3	27	N	137.8	52.9	62	1,067	1,037	NA	NA	Inferior, infero-lateral (basal/medial) and lateral (apical)	6	Epicardial	Y	Y	5

M, male; Y, yes; N, no; LV, left ventricular; LVEDV, left ventricular end-diastolic volume; ECG, electrocardiogram; LVESV, left ventricular end-systolic volume; BSA, body surface are; LGE, late gadolinium enhancement; CAD, coronary artery disease; CA, coronary angiography; CCT, cardiac computed tomography; STE, ST-segment elevation; WMA, wall motion abnormality.

* CMR was performed at 3T scanner, while the rest at 1.5T scanner.

**Figure 1:**
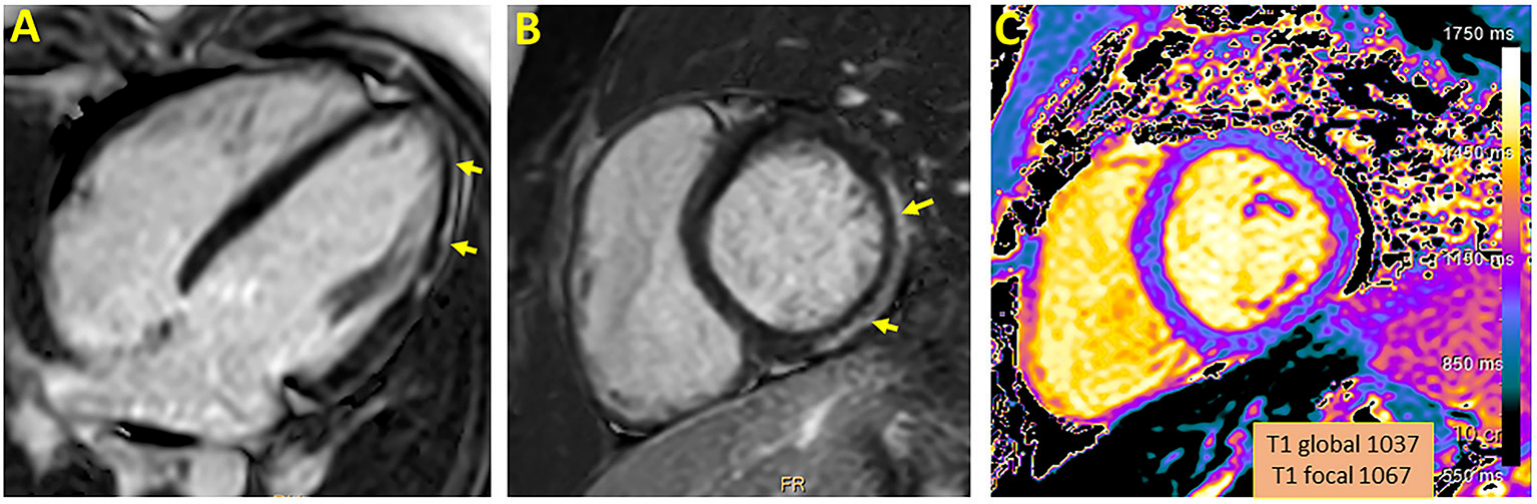
Representative CMR imaging in 4-chamber **(A)** and short axis **(B)** view demonstrating late gadolinium enhancement involving epicardial wall of the inferior and inferolateral segment. A corresponding myocardial injury in native T1 mapping is presented **(C)**.

As presented in Table 1, the median time between the diagnosis to the CMR was 104 days (range 27–149). The median left ventricular ejection fraction, based on CMR, was within normal range 65% (range 62–69%). There were no regional wall motion abnormalities detected in all patients. Native T1 was available in 4 patients, the local native T1 value was increased in three of them. T2 values were available in 2 patients and were all within normal range. The median LGE was 2% (range 0–6%) with inferolateral wall being the most common location (3/5). The patterns of the LGE were as following: (i) mid-wall in 3 patients; (ii) epicardial in 1-patient. LGE in the pericardium was present in 2/5 patients with pericardial effusion present in 4/5 patients with a median diameter of 4 mm (range 3–5 mm) at end-systole. A representative CMR imaging finding is displayed in Figure 1.

## Discussion

The current study reports and elaborate CMR imaging findings, clinical presentation and clinical outcomes of 12–15 year-old adolescents with a clinical diagnosis of myocarditis following the first and second Pfizer BNT162b2 mRNA COVID-19 vaccination in adolescence. This seems to be a rare adverse event (9 out of 182,605 adolescents, 0.0049%) in this group of patients with a male predominance, a mild clinical course and favorable outcomes, which are all consistent with previous initial reports for this and other age groups (1, 2). The CMR imaging findings observed here-in further support the mild and overall benign myocarditis following COVID-19 vaccination, consistent with the CMR findings among older patients (3, 4). In addition, these findings of relatively low LGE%, and its predominant pattern (inferolateral) and normal/near-normal LVEF are known to be strong predictors of favorable long-term outcomes both when the CMR performed at presentation and at follow-up (9), yet this remains to be proven in this particular group of patients.

Limitations of the current study include a relatively small number of patients, lack of confirmation by a myocardial biopsy, CMRs were not performed systematically in all patients and with a non-identical, often delayed interval from diagnosis.

## Conclusions

In conclusion, the CMR imaging findings, consistently with the clinical course, of 12–15 year-old adolescents with vaccine-associated myocarditis following the Pfizer BNT162b2 mRNA COVID-19 vaccination, are similar to those of older patients, being relatively mild and potentially implying favorable clinical course and outcomes of these patients. However, the long-term consequences of this vaccine-associated cardiac myocarditis are not yet fully defined and should be studied.

## Data availability statement

The raw data supporting the conclusions of this article will be made available by the authors, without undue reservation.

## Ethics statement

This study was reviewed and approved by Clalit Health Services institutional review board. Written informed consent was not required for this study in accordance with the local legislation and institutional requirements.

## Author contributions

AS, AH, and RK conceived and planned the study. AS, AH, and AR reviewed the CMR tests. AS and AH contributed to the interpretation of the results and drafted the manuscript. AS, AH, YA, MW, YP, and GW obtained patient related clinical data and contributed to sample preparation and data analysis. All authors provided critical feedback and helped shape the research, analysis, and manuscript. All authors contributed to the article and approved the submitted version.

## Conflict of interest

The authors declare that the research was conducted in the absence of any commercial or financial relationships that could be construed as a potential conflict of interest.

## Publisher's note

All claims expressed in this article are solely those of the authors and do not necessarily represent those of their affiliated organizations, or those of the publisher, the editors and the reviewers. Any product that may be evaluated in this article, or claim that may be made by its manufacturer, is not guaranteed or endorsed by the publisher.
